# Sugarcane biopolymer membrane: experimental evaluation in the middle ear

**DOI:** 10.1590/S1808-86942011000100008

**Published:** 2015-10-19

**Authors:** Débora Lopes Bunzen Mayer, Juliana Gusmão de Araújo, Mariana de Carvalho Leal, Silvio da Silva Caldas Neto, Rafael Figueiredo Ataíde, Roberto José Vieria de Mello

**Affiliations:** 1 MSc. Substitute Professor of ENT at UFPE; 2 3rd year resident in ENT - University Hospital - UFPE; 3 PhD, Head of ENT - Hospital Agamenon Magalhães; 4 Associate Professor - Head of the Medical Residency Program in ENT - UFPE University Hospital; 5 Medical Student at the UFPE; 6 PhD, Associate Professor of Pathology - UFPE University Hospital

**Keywords:** absorbable implants, inflammation, ear, middle, biocompatible materials, tympanoplasty

## Abstract

New developments on biomaterials are important in surgery. The behavior of a new membrane produced from sugarcane will be evaluated in the middle ear of rats.

**Aim:** This study analyzed the results from the interaction of the sugarcane-base biopolymer membrane in the middle ear of a rat.

**Materials and Methods:** We ran an experimental, prospective, paired study with 24 Wistar rats. The sugarcane-base polymer membrane was inoculated in the right ear; and an autologous fascia in the left ear. The rats were divided in 3 groups of 8, and slaughtered at 4, 8 and 12 weeks after surgery. Histological analyses were performed on the rats' middle ear mucosa and their tympanic membranes.

**Results:** There was an inflammatory reaction on the experimental group and middle ear subacute exudate in 50%of the cases; 30% chronic exudate; and 20% was normal. In the control group there was only one case of exudate. The inflammation was initially described as intense, but it decreased over time. Myringosclerosis was observed in both groups. The sugarcane biopolymer membrane was absorbed later when compared with fascia.

**Conclusion:** The sugarcane biopolymer membrane induced an inflammatory reaction in the middle ear which decreased over time, and mild fibrosis. Future studies can indicate its use in otolaryngology.

## INTRODUCTION

The materials used in surgery can be classified into biological and synthetic, according to their origin (either organic or not)^1^. The biological material can be autologous - when taken from the same individual; homologous - when taken from other individuals of the same species, or heterologous - tissue from another animal species^2^. The main ear surgeries requiring biomaterials are those used to repair tympanic membrane perforation (MT) and for ossicular chain reconstruction.

The autologous materials used for grafting are fat tissue, perichondrium, cartilage, dura mater, periosteum from the temporal bone and fascia^2^, ^3^, ^4^. The temporal muscle fascia is one of the most used autologous grafts^5^. One limiting factor to the use of autologous materials is the difficulty in using them in revision surgeries^2^.

Thus, other types of materials are being considered to be used as graft, such as support material or as healing adjuvants for the surgical wound in middle ear surgery. The biocompatible materials used to replace human tissue or to help heal wounds cause some degree of inflammation. Such inflammation, however, must not be very intensive, but enough to cause tissue repair.

Epidisc^®^ and the Epifilm^®^ are membranes made up from an ester of hyaluronic acid, however with poor results when used as graft material in tympanoplasty^6^. Membranes made from the silk protein, from the bladder tissue matrix and the swine intestine are among the new proposals^7^, ^8^, ^9^. Substances developed from molecular biology, such as the growth factors present in the inflammatory phase of the TM healing are also being tested^10,^^11^.

Within this concept of new biomaterials we have the biopolymer. A biopolymer is a macromolecule made up of numerous monomers. The polymers which derive from vegetal oils, such as latex, are already being tested in human beings. In ear surgery, one latex membrane from the rubber tree was used to coat the surgical neocavity of the canal-wall-down tympanomastoidectomy and caused early wound healing^12^.

The sugar cane biopolymer is made up of numerous exopolysaccharides, 87.6% is glucose. The polysaccharides are synthetized and excreted by the *Zoogloea sp.*, when this bacteria is within a medium rich in sugar cane syrup^13^.

The biopolymer *in natura*, with residual sugars, was used as dressing in the treatment of accidental injuries in dogs. The dressings caused: increased in granulation tissue, infection control, and shorter healing time^14^. Afterwards, this biopolymer was purified and processed, resulting in a homogeneous, stable tissue, with low toxicity and physicochemical characteristics which meet the morphofunctional specificities of different tissues^15^.

Brazil is the largest sugar cane producer in the world. The possibility of developing a new biocompatible material, which can be used in ear surgery, produced from a raw material which is abundant in our country has encouraged us to run the present study.

## MATERIALS AND METHODS

We used twenty-four albino, male Wistar rats (*Rattus norvegicus albinus*), weighing 335 to 440g (mean weight of 392g), with about four months of age. The study design was: experimental, prospective and paired. As exclusion criterion we considered any middle ear change or TM change (perforation or tympanosclerosis).

In the ears of the experimental group (right side), the TM was perforated in its *pars tensa*, with the introduction of a strip of the sugar cane biopolymer membrane measuring 3mm in length and 1 mm in thickness. In the control group ears (contralateral left side), the autologous abdominal fascia was introduced with the same dimensions of the polymer membrane, in the same shape as per described for the experimental group.

The rats were randomly broken down into two groups of eight animals in each: group t1 (slaughtered after 4 weeks of surgery), group t2 (slaughtered after 8 weeks) and group t3 (slaughtered after 12 weeks). After slaughtering, the tympanic bullas were submitted to histochemical processing with decalcification and inclusion in paraffin. The slides were dyed with hematoxylin and eosin (HE). In some slides we used the Picrosirius Red (PSR) besides the HE.

Histocompatibility was assessed by an experienced pathologist, and had the following criteria: the inflammatory reaction intensity caused by the presence of fibrosis in the tissue in contact with the material. We observed signs of inflammation and fibrosis in the mucosa and the TM. We also investigated the likelihood of the sugar cane polymer membrane be absorbed in the middle ear of the rats.

The type of inflammation was divided into acute, subacute and chronic. The acute histological pattern is characterized by the exudation of liquid and plasmatic proteins (edema) and the migration of leucocytes, especially neutrophils. The chronic pattern is characterized by the presence of lymphocytes, macrophages, blood vessel proliferation, fibrosis and tissue necrosis. The subacute type is defined by the inflammatory reaction with components of the acute and chronic patterns occurring simultaneously.

Some semi-quantitative criteria were adopted. The intensity of the inflammatory process was described based on the finding of cellularity in the exudate, on the extension of the process through the tympanic bulla and by the presence of cell necrosis. The inflammatory activity intensity was classified as follows: nil - no signs of inflammation, mild exudate with little cell infiltrate, reaction involving up to 1/3 of the bulla lumen, no necrosis; moderate - exudate with moderate cell infiltrate, reaction involving between 1/3 and 2/3 of the bulla lumen, a little necrosis; intense - exudate with large cell infiltrate, reaction involving more than 2/3 of the bulla lumen, intense cell necrosis.

Fibrosis is characterized by the presence of fibroblasts and collagen deposits on the extracellular matrix. In order to confirm the presence of fibrosis in some slides selected by the pathologist, besides HE dyeing, we also used PSR dyeing. Subepithelial bulla fibrosis was classified as mild, moderate and intense.

In assessing the degree of absorption of the tympanic bulla material: nil - material without signs of absorption; mild absorption - peripheral fragmentation with cellular infiltration in the material; moderate absorption - central fragmentation with a cell infiltrate; intense absorption - total fragmentation of the material; complete absorption - no material.

The present study was approved by the Ethics Committee in Animal Experimentation of the Center of Biology Sciences, where the experiment was registered under protocol # 23076.019069/2008-11.

## RESULTS

### Control group descriptive results

The mucosal ciliated epithelium was preserved or with mild edema in 18 cases. Nonetheless, in two ears of group t1 and in four from group t2 there was subepithelial edema, with unspecific inflammatory reaction. We did not notice fibrosis.

The TM was histologically normal in most of the ears studied, or with mild thickening in the intermediate layer. Signs of tympanosclerosis were seen in six ears.

**Figure 1:**
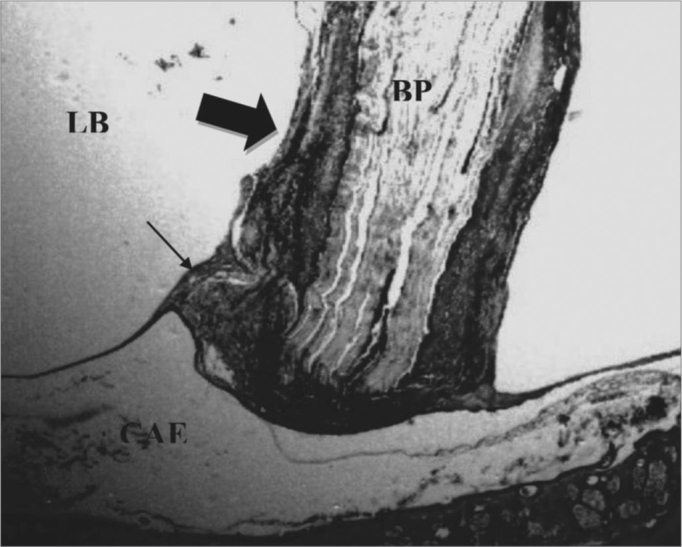
Control Group. Microphotography of the fascia adhered to the tympanic bulla mucosa. LB= bulla lumen; OT = Temporal bone; thin arrow= fascia. HE (100x magnification).

In the lumen of the tympanic bulla we found one case with exudate during t1 time, considered moderate. The fascia was found in only four ears, two in t1 and one in each of the other times (Figure 1).

### Experimental group results

**Figure 2:**
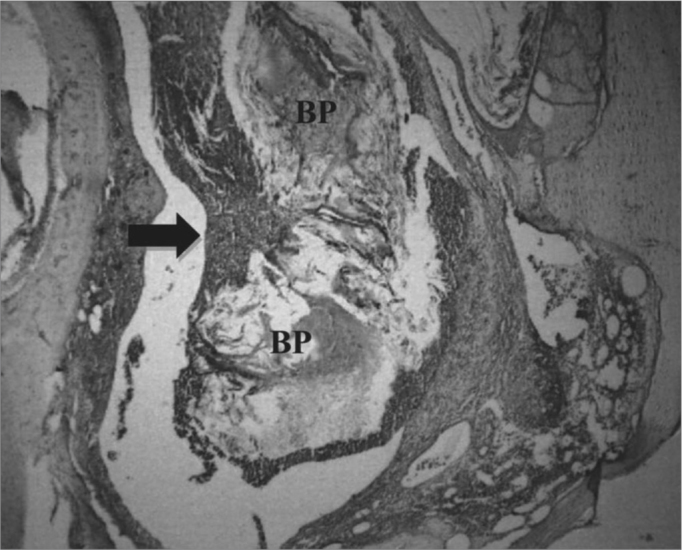
Experimental group with 4 weeks - Microphotography of the subacute exudate of moderate degree around the sugar cane biopolymer membrane; BP= sugar cane biopolymer. N= cell necrosis. HE (100x magnification)

**Figure 3:**
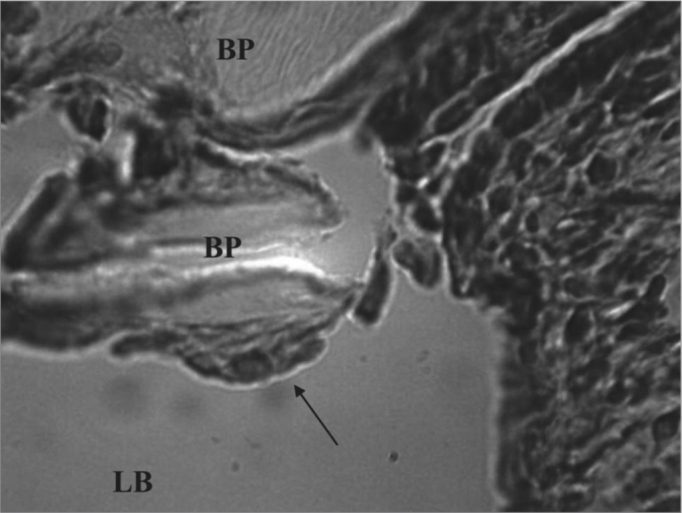
Experimental group with 12 weeks - Microphotography of the sugar cane biopolymer membrane; on the tympanic bulla without signs of inflammatory reaction. OT= temporal bone; broad arrow = sugar cane biopolymer. HE (40x magnification).

There were inflammation signs in the tympanic bulla, with characteristics of subacute and chronic reaction. We did not find changes which would indicate an exclusively acute inflammatory process. There was a subacute exudate in approximately 50% of the cases, 30% chronic exudate and in 20% of the cases we did not find any reaction whatsoever (Figures 2 and 3).

The subacute inflammatory process was seen in 11 ears (Figure 2). There were five cases in t1 and six in t2. The subacute reaction intensity was described as mild in three cases, moderate in five and intense in three cases, according to the semiquantitative criteria adopted.

**Figure 4:**
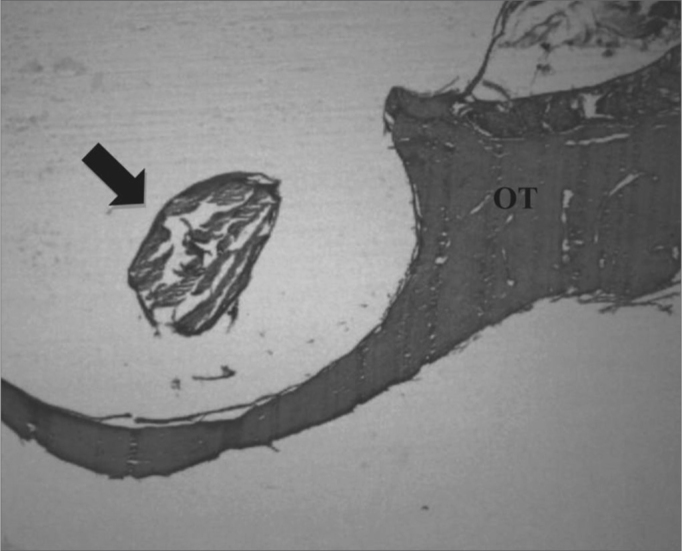
Experimental group chronic reaction - Microphotography of a sugar cane biopolymer membrane having been engulfed by giant cells; foreign body type; BP= sugar cane biopolymer. LB= bulla lumen. Thin arrow =giant cell. HE (1000x magnification).

Chronic inflammation was found in six ears (three cases in t1, one in t2 and two in t3). The presence of giant cells characterized this reaction as foreign body type (Figure 4). The chronic reaction intensity was mild in all the cases.

**Figure 5:**
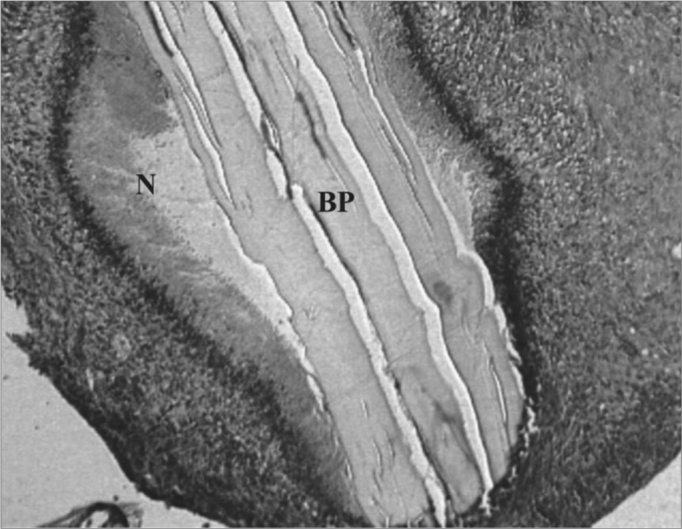
Experimental group at 8 weeks - Microphotography of the tympanic membrane, thickened at the point of contact; sugar cane biopolymer. Surrounded moderate subacute exudate; the biomaterial. BP= sugar cane biopolymer; LB= bulla lumen; CAE= external ear canal; thick arrow = inflammatory reaction; thin arrow =malleus. HE (40x magnification).

The ciliated columnar epithelium was preserved or it had mild edema in 16 ears from the experimental group. Mucosal changes were localized and did not extend to the entire air space of the tympanic bulla; nonetheless, the point of direct contact with the material had the most reactivity (Figure 5).

The TM remained normal or with mild thickening in 13 ears. The histological changes seen pointed towards tympanosclerosis of varied degrees.

As far as the fibroblastic activity goes most of the cases were histologically classified as mild fibrosis. There was a greater thickening on the mucosa, with collagen deposits seen upon PSR dye, in two ears only, one in t1 and another in t2.

### Analytical results

On Table 1 we see a comparison of the bulla exudate with the biopolymer and the fascia, with statistically significant differences between them.

**Table 1:** Assessing the inflammatory activity intensity according to the type of material.

Inflammatory activity	Fascia	Biopolymer	Total	p Value
Nil	23	7	30	p(1) < 0,001*
Mild	-	9	9	
Moderate	1	5	6	
Intense	-	3	3	
Total	24	24	48	

(*): Significant difference at 5.0%. (1): Through Fisher's Exact Test.

**Figure 6:**
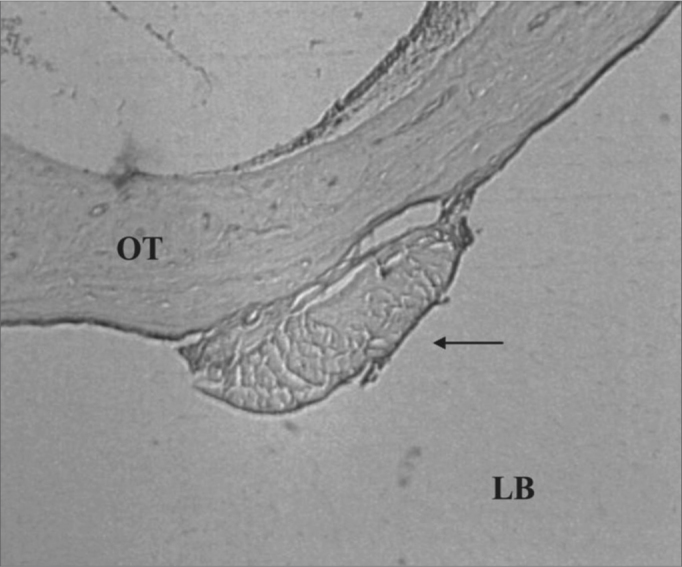
Signs of biomaterial absorption - Microphotography of the membrane's structural disorganization; sugar cane biopolymer; and its partial absorption, seen in the tympanic bulla at 8 weeks; BP= sugar cane biopolymer; thick arrow = inflammatory exudate. HE (40x magnification)

The material absorption analysis can be seen on Table 2. Notice that the sugar cane polymer membrane degraded with time, with intense fragmentation in two cases of t2 (Fig. 6) and three cases of complete absorption in t3.

**Table 2:** Assessing the degree of absorption according to the type of material.

Degree of absorption classification	Fascia	Biopolymer	Total	p Value
Nil or mild	4	15	19	p(1) < 0,001*
Moderate	-	4	4	
Intense	-	2	2	
Complete	20	3	23	
Total	24	24	48	

(*): Significant difference at 5.0%. (1): Through the Fisher's Exact test.

On Table 3 we see only the experimental group and the degree of inflammatory activity along time. There was a statistically significant difference between the times of the experiment with exudate reduction on t3.

**Table 3:** Assessing the intensity of the inflammatory exudate in the experimental group.

Time of the experiment					
Inflammatory intensity	T1	T2	T3	Total	P value
Nil	-	1	6	7	p(1) = 0,026*
Mild	5	2	2	9	
Moderate	2	3	-	5	
Intense	1	2	-	3	
Total	8	8	8	24	

(*): Significant difference at 5.0%. (1): Through Fisher's Exact Test.

## DISCUSSION

The sugar cane biopolymer membrane was initially used in ear surgery as a free lateral graft in chinchillas. In this situation, there was a TM perforation closure similar to what was found in the control group^16^. In the current study, the membrane was placed medial to the TM with the aim of assessing its behavior on the middle ear mucosa. In order to do that, we compared it with the fascia, which is the autologous material most frequently used in ear surgery. There was inflammatory reaction with exudate in most of the cases in the experimental group, with statistically significant difference seen in the autologous fascia group (Table 1).

The rat's tympanic bulla is anatomically and histologically similar to the human middle ear^17^. The study of the reactions materials have in the tympanic bulla of rats is important, since it enables to predict the behavior of the middle ear mucosa of human beings in comparison to different substances.

We know that this mucosa reacts with an intense inflammatory response to most aggressor agents. One study was carried out injecting inert substances, pathogenic and non-pathogenic substances in the middle ear of rats. The authors did not find important differences as far as the inflammatory response intensiveness goes in relation to each substance, except when there was a live bacteria^18^. It was then concluded that regardless of the type of substance in contact with the rat's ear mucosa, the latter is highly reactive, and there is the possibility of causing otitis media^18^.

The exudate seen around the sugar cane biopolymer membrane matches the one observed in other semisynthetic materials. Papers comparing the biopolymer membrane with e-PTFE and polypropylene have shown the inflammatory reaction in a similar intensity^19,^^20^. The sugar cane biopolymer membrane caused an inflammatory reaction that was more intense than that of the fascia, an autologous biological material. It may be that if we had compared the biopolymer with another biomaterial the results would have been different.

The TM's inner layer is contiguous with the middle ear mucosa and reacts to the same stimuli. Tympanosclerosis was present in both groups. It is likely that the TM thickening was secondary to its perforation. One can insert material in the tympanic bulla through the retroauricular access, with later bone trephination, nonetheless, we chose a pathway with less tissue trauma and one that is technically faster. Should there be a third group submitted only to perforation, but without material inoculation, we could better identify whether TM changes could result from contact with the material or the surgical manipulation.

Inflammation reduced over time (see Table 3). At the end of the experiment, the tympanic bulla recovered from its initial reaction, and there was no exudate and a little fibrosis. Despite the moderate/intense inflammatory reaction being found in 33% of the cases, the sugar cane biopolymer membrane apparently caused less fibrosis than what was reported from the Gelfoam^®^®^21,^^22^material. One support material to the graft, and it remains in the proper place until the tissue heals, without causing middle ear fibrosis or adherences.

One study on a new material for the pubovaginal sling compared the sugar cane biopolymer with polypropylene and divided the animals in two groups of 30 and 90 days^19^. As a result, the author found an intense inflammatory reaction with 30 days and mild inflammatory reaction with 90 days. Such data is similar to what was found in the present study, when after 12 weeks (90 days) we noticed a reduction in the inflammatory activity.

On Table 2, we see that the fascia was absorbed much more easily than the biopolymer, and such difference was statistically significant. Fascia is a live tissue and needs nutrition to maintain itself. In the bulla lumen it did not find the ideal conditions to maintain itself, it very likely necrosed and was absorbed.

The sugar cane biopolymer membrane is an inert material, and was kept intact in most of the cases. Nonetheless, it degraded as of t2 time, with its absorption in the three cases in time t3. The capacity of being absorbed or replaced in the open bed was observed when the biopolymer membrane was used to replace the dura mater of rats. In this situation, there was a propensity to absorption, with signs of disorders in the original architecture and its replacement by fibrosis^20^.

One important characteristic of biomaterials is its capacity to integrate with live tissue. The polylisin latex membrane, a type of biopolymer, was experimentally used in dogs to correct esophageal defects. The biomembrane caused tissue scarring, with spontaneous shedding from the open bed as soon as there was complete neoformation on the extension covered by it^23^. The sugar cane biopolymer apparently is not expelled, but rather absorbed by the tissue. One future possibility would be to assess its integration as a graft or as prosthesis in the middle ear.

The sugar cane biopolymer membrane caused an inflammatory reaction in the rats' middle ear, with greater intensity that that of the autologous fascia. Other studies need to be carried out in order to check the possibility of its use in ear surgery, as graft material in tympanoplasty, support material or even to make prosthesis for ossicular reconstruction.

## CONCLUSION

The sugar cane biopolymer membrane caused an inflammatory reaction in the middle ear of rats with remission in the later time of the experiment. The likelihood of its future use in ear surgery still requires further studies.
